# MicroRNA-656-3p inhibits colorectal cancer cell migration, invasion, and chemo-resistance by targeting sphingosine-1-phosphate phosphatase 1

**DOI:** 10.1080/21655979.2022.2031420

**Published:** 2022-01-26

**Authors:** Baoming Zhang, Shanting Gao, Zengtao Bao, Cheng Pan, Qingshui Tian, Qiang Tang

**Affiliations:** Gastrointestinal Department, The First People’s Hospital of Lianyungang, Lianyungang City, Jiangsu Province, China

**Keywords:** Colorectal cancer (CRC), miR-656-3p, SGPP1, 3’ UTR of *SGPP1*, metastasis

## Abstract

Colorectal cancer presents high rates of recurrence and metastasis, and the occurrence and progression and mechanism of its invasion and metastasis are not fully understood. The expression of miR-656-3p in patient samples and 10 cell lines were measured. Bioinformatic databases were used to predict miRNAs. Protein expressions were examined using Western blot. Transwell assay was used to measure cell migration and invasion. Transplanted tumor model in nude mice was established. Removal of the miR-656-3p by specific knocking-down of this gene promoted the chemo-resistance of colorectal cancer cells. Critically, we identified sphingosine-1-phosphate phosphatase 1 (SGPP1) as a downsteam target of the miR-656-3p, which we first obtained from 199 potential target genes from Targetscan, 200 genes from miRDB and 200 genes from DIANA, respectively. Then, we identified the interaction between SGPP1 and the miR-656-3p on 3’ UTR of SGPP1 gene. Knockdown of SGPP1 greatly suppressed the tumor growth in vivo and epithelial mesenchymal transition process. miR-656-3p could regulate cell proliferation and chemoresistance in the colorectal cancer that associate to downstream target with SGPP1. Along with its downstream molecule, we would like to predict that the SGPP1 associated miR-656-3p could be used to develop early for early diagnostics for CRC oncogenesis.

## Graphical Abstract

**Figure:**
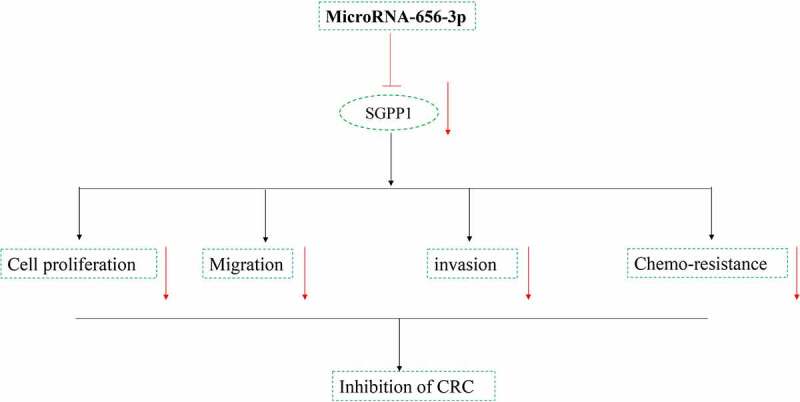

## 1. Introduction

As a common cancer within gastrointestinal tract, colorectal cancer (CRC) is known as the third largest cohort and the second most common malignancy in women worldwide [1]. The CRC incidence is trending strongly upward and became as the fourth leading cause of malignancy and death in China with high rates of recurrence and metastasis are the major causes of death in CRC patients [2]. As the 5-year survival of early stage CRC is higher after surgery, the long-term survival rate and prognosis of advanced CRC are poor [3]. The occurrence and progression of and the mechanism of its invasion and metastasis was not yet fully illustrated because of multiple factors, stages, steps, and genes involved in the mechanism [4,5]. Current studies demonstrated that the changes related to occurrence, development, invasion, and metastasis associated with oncogenes, tumor suppressor and tumor metastasis suppressor genes. Besides oncogenes including C-myc and Her-2, tumor suppressors such as p53 and phosphatase and tensin homologue (PTEN) were deleted in colorectal carcinoma (DDC) along with tumor metastasis suppressor gene *nm23*. Recently, extracellular matrix proteins such as MMPs, CD44 and mucins (MUC), DNA mismatch repair (dMMR) deletion, KRAS and BRAF mutation are all linked to CRC oncogenesis [6,7].

MicroRNA as a type of small non-coding RNA (ncRNA) contains 19–24 nucleotides plays many functions of regulation in many biological processes pathologically [6,7]. Mature miRNAs formed through a series of nuclease digestion of longer premicroRNAs, can recognize mRNA with the 3ʹUTR region and then degrade mRNA or block the translation of mRNA [8], which could be a key function in the occurrence and development of malignancy. Abnormally expression of miRNAs is involved in the occurrence of several categories of cancers including breast cancer [9], lung cancer [10], bladder cancer [11], and colon cancer. MicroRNA-214-3p modified tetrahedral framework nucleic acids target survivin to induce tumor cell apoptosis [12]. MicroRNA-301b-3p contributes to tumor growth of human hepatocellular carcinoma by repressing vestigial like family member 4 [13]. MicroRNA-551b-3p inhibits tumor growth of human cholangiocarcinoma by targeting Cyclin D1 [14]. MicroRNA-1323 downregulation promotes migration and invasion of breast cancer cells by targeting tumor protein D52 [15].

As well know that, micro RNA (miRNA) take a role of tumor suppressor or oncogene during the process of cancer. It has been reported that miR-656-3p was significantly under-expressed in the cancer tissues of nonsmall cell lung cancer patients and it also showed the inhibition function in the proliferation and migration of non-small cell lung cancer cell lines [16]. Sphingosine-1-phosphate phosphatase 1 (SGPP1) has been reported to be closely related with the regulation of several kinds of tumors. miRNA-95 mediated radioresistance in tumors by targeting the sphingolipid phosphatase SGPP1 [17]. SGPP1 was involved in the inhibition of cell viability and promotion of cell apoptosis in colon cancer by sevoflurane [18]. microRNA-27a suppressed colorectal carcinogenesis and progression by targeting SGPP1 [19].

In this study, we hypothesized that miR-656-3p might inhibit the migration, invasion, and chemo-resistance of CRC. We aimed to investigate if miR-656-3p could affect CRC through targeting SGPP1. We detected the low expression of miR-656-3p in 119 colorectal cancer tissues by qRT-PCR. And through lentivirus transfection, double luciferase analysis, cell proliferation and migration/invasion examination, and cell chemoresistance detection, we further approved that miR-656-3p inhibited the proliferation and migration of colorectal cancer cells by directly targeting to Sphingosine-1-phosphate phosphatase 1 (SGPP1). The result provides strong potential for developing miR-656-3p- SGPP1 as treatment against colorectal cancer.

## 2. Methods and materials

### 2.1 Collection of clinical samples

This study was approved by ethics committee of First People’s Hospital of Lianyungang. All human studies in this paper were performed according to the principles of Declaration of Helsinki. All patients included in our study were fully informed with the details and process of this study, and the informed consents were provided. 119 CRC patients (Table 1) without any previous treatment, including radiation, chemotherapy and immunotherapy treatment, were enlisted from the First People’s Hospital of Lianyungang. All diagnosis of CRC was confirmed by at least two senior oncologists. CRC tissues and tumor adjacent normal tissues of each patient were sampled and kept in liquid nitrogen immediately for RNA extraction. The total RNA were kept at −80°C before using.

**Table 1.** Clinical features of the patients

Features	Total(n)	*p* value
Gender		
Male	62	0.613
Female	57	
Age (years)		
≥60	39	0.592
<60	80	
T Stages		
I–II	75	<0.001
III–IV	44	
Metastasis		
N Stages		
N0	59	<0.001
N1-2	60	
M Stages		
M0	72	<0.001
M1	47	

### 2.2 Cell culture and treatment

NCM460, SW480, RKO, DLD1, SW48, HCT116, LOVO, HT29, HCT8, and SW620 cell lines were obtained from ATCC (VA, USA). All the cell lines were maintained in corresponding medium including RPMI 1640 and DMEM medium (Gibco, CA, USA) with 10% fetal bovine serum (FBS, Invitrogen),100 U/ml penicillin and 100 μg/ml streptomycin. All the cells were cultured in a humidified incubator with 5% CO_2_ environment at 37°C.

### 2.3 Target genes knockdown and overexpression

The inhibitors targeting the *miR-656-3p* used in this study is double-stranded siRNAs (dsRNA), which were synthesized by Chemical Methods (ReiBo Biotech, Guangzhou, China). The siRNAs targeting the SGPP1 were purchased from ReiBo Biotech as well. SW480 cells were selected as the knockdown cell model. The cells were seeded at a density of 5 × 10^5^ cells per well in 6-well plates and cultured in DMEM supplemented with 10% FBS (without streptomycin and penicillin) for at least 12 hours. OPTI-MEM serum-free medium (Gibco, CA, USA) and Lipofectamine 2000 reagent (Invitrogen, CA, USA) were used for the transfection. The final concentration of siRNA was set at 100 nM level. After transfecting for 24 hours, the cells were collected for cell counting assay and protein extraction.

The miR-656-3p mimics and their negative control purchased from RiboBio (ReiBo Biotech, Guangzhou, China) were conducted for miR-656-3p overexpression study. 200 nM miR-656-3p mimics and their negative control were separately transfected to the DLD1 cells (with 30–50% confluence) by Lipofectamine 2000 in media (Thermo Fisher Scientific, USA), based on the protocol provided by the manufacturer. The transfected cells were cultured for 48 hours in a 5% CO_2_, 37°C incubator. The sequences were listed as follows: miR-656-3p mimics: ACGUCACUGCGUUCUCUCCCCG, NC mimics UACUTCGAACCAACCAGCGUUACT; miR-656-3p inhibitor CGGGGAGAGAACGCAGUGACGU, NC inhibitor CATUCCUUGTUGUGUAAUCCUUTT; SGPP1siRNA-1: 5ʹUUAUACUGCGUGAGUGCACTT3’, 5ʹTTAAUAUGACGCACUCACGUG3’. SGPP1siRNA-2: 5ʹUUACCUUUCGGUCACACCCTT3’, 5ʹTTAAUGGAAAGCCAGUGUGGG3’. SGPP1siRNA-3: 5ʹUUACUCGUGUCCUGUCAACTT3’, 5ʹTTAAUGAGCACAGGACAGUUG3’.

### 2.4 Total RNA extraction and real time reverse transcription polymerase chain reaction

Total RNA prepared for real-time reverse transcription polymerase chain reaction (real-time RT-PCR), was extracted from clinical sample and cultured cells with MiniBEST Universal RNA Extraction Kit (TaKaRa, Japan) and then stored in −80°C for future use. To quantify miR-656-3p, the complementary DNA (cDNA) was synthesized with PrimeScript™ RT reagent kit from TaKaRa (Dalian, China). The miR-656-3p-specific stem-loop, RORA, ITGB8, RGCC, and SGPP1 RT primers was purchased from RiboBio (Guangzhou, China), U6 primers was from Cell Signaling Technology (Danvers, USA). The primer sequences are as follows: miR-34a-5p forward, 5’- CTGGGAGGTGGCAGTGTCTTAGC- 3’ and reverse, 5’- TCAACTGGTGTCGTGGAGTCGG −3’; RORA forward, 5’- CTTGCCGTAGGGATGTCTCG −3’ and reverse, 5’- GAAGTTCCGTCAGCCCGTT −3’; ITGB8 forward, 5’- GTGAAAGTCATATCGGATGGCG −3’ and reverse, 5’- GCTATCAAGAGCGAGATGAGACG −3’; RGCC forward, 5’- CGCCACTTCCACTACGAGG −3’ and reverse, 5’- CAGCAATGAAGGCTTCTAGCTC −3’; SGPP1 forward, 5’-TGGTCCTCCTCACCTATGGC-3’ and reverse, 5’-CTAGAGAACACCAGCAGGGA-3’; U6 forward, 5’-GTGACCTTTATTGCG ACATCCACT-3’ and reverse, 5’-CTTCTGAAACACGAGTCATATGTGGT-3’.

The 10 μL reacting system contained 2 μL of 5× RT buffer, 0.5 μL of PrimeScriptTM RT Enzyme Mix, 1 μL of miR-656-3p RT/SGPP1/ RORA/ITGB8/RGCC primer,1 μL of total RNA, and 5.5 μL of sterilized water. The reacting system was treated at 37°C for 15 minutes, 85°C for 5 seconds, and then the cDNA template were synthesized. The following qRT-PCR procedure was completed with Bio-RadIQ5 real-time PCR system and SsoFast™ EvaGreen Super blend pack (Bio-Rad). The reacting system contained 10 μL of SsoFast™ EvaGreen super blend, 1.5 μL of forward primer and reverse primer from RiboBio, respectively, 2 μL of cDNA template, and 5 μL of sterile water. The program was set as follow, 95°C for 30 seconds, then 40 cycles of 95°C for 5 seconds and 60°C for 10 seconds. U6 was performed as the internal control to normalize the expression of each target genes. The relative expression of each target genes were then calculated with the 2-ΔΔCt cycle threshold method.

### 2.5 Scratch-wound assay

All treated cells for scratch-wound assay were resuspended and seeded. After transfection, the scratch test was performed with alcohol and inserted in a 12-well plate with the arrangement of three replicates per group. The resuspension cells were diluted to a concentration of 500 cells/μL with medium, and seeded to 12-well plate with 70 μL per well then placed in a 37°C incubator filled with 5% CO_2_ for 24 hours. After 24 hours, the cells were gently scratched with a pipette tip across the diameter and then washed twice with PBS and then replaced with 1 ml fresh medium supplied with 1% FBS (0 hours) for another 24 hours culture. The status of the cells was observed and documented at 0 hour and 24 hours with Ob-A1 Zeiss microscope. The percentage of each healing group was calculated as described in publication [20].

### 2.6 Migration and invasion assay

Transwell Porous Back (Corning Consolidated, Corning, NY, USA) was applied for the cell migration test. Cells were carefully moved to the beat chamber of at a thickness of 1 × 10^6^ per ml. After incubating for 24 hours, the emigration of the cell on the transwell was observed and quantified. Cells reached the foot side of the film were fixed in methanol, stained with hematoxylin and then counted.

The invasion of the cell was tested with Cultrex 24-well BME Cell Invasion Assay kit (Trevigen Inc., MD, USA), based on the manufacturer’s instruction. In short, 1 × 10^3^ differently treated cell were implanted into the upper wells precoated with Matrigel storm cellar extricate containing 100 µl serum-free media (Trevigen Inc., MD, USA). Then, 500 µl of fresh harvested cells was implanted into the bottom wells. The cells migrated to the lower surface were fixed with 500 µl of Cell Dissociation Solution (Calcein-AM). The fixed cell were incubated at 37°C in CO_2_ for 1 hour and quantified with fluorometric analysis.

### 2.7 Cell viability analysis

Cells were plated in 6-well plates with a density of 10^5^ cells and then cultured for 24 hours in CO_2_ at 37°C. The cells were treated with 5-Fluorouracil (5-FU) in a dose of 0, 10, 20, 30, 40, 50, 60, 70, and 80 g/ml, then further incubated with the tetrazolium salt, 3-(4, 5-dimethylthiazol-2-yl)-2, 5-diphenyltetrazolium bromide (MTT, 0.5 mg/ml) for 3 hours at 37°C. Formazan crystals were solubilized with acidic isopropanol (40 mM) and the absorbance was measured through a Victor 3 microplate reader (Perkin Elmer, Finland) at 570 nm [21,22].

### 2.8 Western blot analysis

The protein of each cells groups was extracted with a RIPA Lysis and Extraction Buffer with 1% PMSF (Beyotime, Shanghai, China), and the total protein concentrations were normalized then mixed with 4x protein loading buffer (Invitrogen, CA, USA). Sodium dodecylsulfate–polyacrylamide (SDS-PAGE) buffer was applied to separate the total protein. The separated proteins were then transferred onto a polyvinylidene difluoride film (Novus, CO, USA), followed by blocking with 5% nonfat milk in TBS-T for 1 hour at room temperature, and incubated in primary antibody for 12 hours at 4°C. The membranes were incubated with the secondary antibodies for 1 hour at room temperature. The proteins were detected with ECL Chemiluminescence Detection Kit (PromoCell, German). The results of Western blot (WB) were observed with Chemiluminescence Imaging (Clinx Ltd., Shanghai, China). The primary antibodies used in this study include: anti-Rabbit SGPP1 (1:500) (#ab129253, Abcam, MA, USA) and GAPDH (#14 C10) Rabbit mAb (1:1000) (#2118, CST, MA, USA). The secondary antibodies used in the study including anti-rabbit IgG (H + L) (Cat#4414, CST) and anti-mouse IgG (H + L) (Cat#4410, CST) were obtained from CST as well.

### 2.9 Dual fluorescein reporter gene analysis

The renilla luciferase (RLU) internal control plasmid and mimics were transfected into different groups. The cell culture medium was drained After 24 hours, removing the cells medium and lysing the cells fully based on the amount illustrated in the manufacturer’s manual of Dual Luciferase Reporter Assay Kit (Thermo Fisher, USA). The mixture was centrifuged at 10,000 G for 5 min and then 100 μL of the supernatant was performed for quantification. The RLU value quantified by the Renilla luciferase assay was used to separate the RLU value measured by the firefly luciferase measurement. Activation level of the reporter genes for different sample was compared based on the ratio obtained above.

### 2.10 Bioinformatics analysis

TCGA (https://www.cancer.gov/) database were used to analyze the expression of miR-656-3p and its targeting gene in CRC or normal tissues. ICGC (https://dcc.icgc.org/releases/current/Projects) was used to predict the overall survival probability and risk level. miRDB (http://mirdb.org/), DIANA (http://diana.imis.athena-innovation.gr/) and TargetScan (www.targetscan.org) databases were used for predicting miRNA targets.

### 2.11 Transplanted tumor animal model

HT29 cells (5 × 10^5^) suspended with 0.1 mL PBS were subcutaneously injected into the mammary fat pad of nude mice. The animals were subcutaneously treated with SGPP1 siRNA (5 µM, 0.05 mL) or same amount of PBS every 3 days for 3 weeks. Then, the mice were sacrificed and tumors were collected. The tumor weights were calculated and the tumor tissues were used for immunohistochemical staining. The experiments have been approved by the Animal Care and Use Committee of The First People’s Hospital of Lianyungang.

### 2.12 Immunohistochemical staining

The tumor tissues were de-paraffined firstly. After treatment with gradient ethanol, hydrogen peroxide, antigen repair, blocking, and PBS washing. Then, the sections were cultured with primary antibodies overnight at 4°C. The PBS was used to wash slides, and second antibody were used to incubate sections for 2 hours at room temperature. DAB regent was applied to incubate sections, and the protein expression was observed using an inverted microscope. Image J software was used to analyze protein expression. The antibodies were listed as follows: mouse monoclonal to E Cadherin (#ab1416), mouse monoclonal to N Cadherin (#ab19348), mouse monoclonal to Snail (#ab224731), goat anti-mouse IgG (#ab205719).

### 2.13 Statistics

SPSS16.0 statistical software was used to performed the statistical analysis in this study. The data were presented as χ ± s. Both groups were compared with *t*-test. Variance was studied with one-way analysis to compare both groups. pi values < 0.05 were set to have a significantly statistical difference. The experiments in this study were performed at least three independent times before data analysis.

## 3. Results

### 3.1 Omni-expression of miR-656-3p in patients with colorectal cancer indicates its critical function

**Figure 1.**
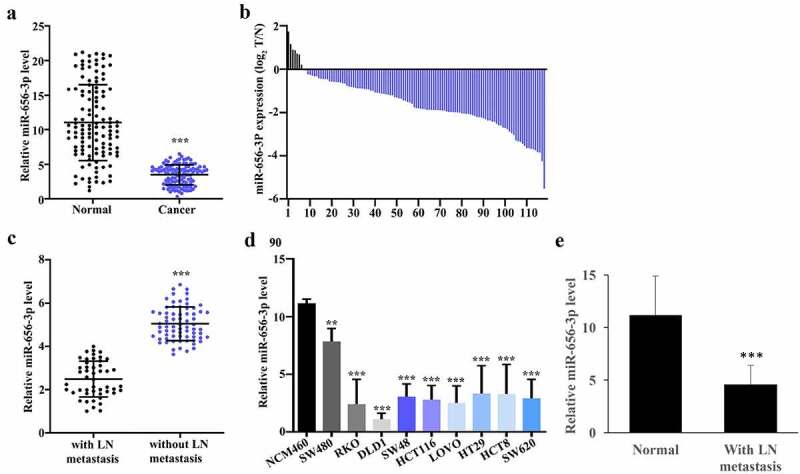
The expression of miR-656-3p is downregulated in CRC tissues. (a) Abundance analysis of miR-656-3p in 119 tumor and their paired cancer-free tissues. (b) The distribution analysis of the miR-656-3p expression in tumor and in adjacent tissues among 119 pairs of specimens. (c) Abundance analysis of miR-656-3p in cancer tissues with or without lymphatic metastasis. miR-656-3p was upregulated expression in colorectal cancer tissues without lymphatic metastasis. (d) miR-656-3p expression in NCM460, SW480, RKO, DLD1, SW48, HCT116, LOVO, HT29, HCT8, and SW620 cells. U6 was employed as an internal reference. (e) The level of miR-656-3p was measured in the normal tissues and without LN metastasis tissues. **p < 0.01, ***p < 0.001 compared with group NCM460.

By applying quantitative RT-PCR analysis on total RNAs extracted from 119 pairs of colorectal cancer tissues (n = 119) and cancer-free normal tissues (n = 119), we found that when in comparison with cancer-free normal tissues, miR-656-3p in CRC tissues is significantly low expressed with *p* < 0.001 (Figure 1(a)). And by further comparing the miR-656-3p expression ratio of CRC and its paired normal cancer-free tissues, we got the fold change of miR-656-3p in each CRC patient (Figure 1(b)). The result showed that, only in few CRC patients (n = 7), the miR-656-3p in CRC tissues presented higher expression than normal cancer-free tissues. Most patients (n = 112) have the lower expression of miR-656-3p in CRC tissues than normal tissues, among these, in more than 51% patients, the expression of miR-656-3p is four to even 30 times less than normal cancer-free tissues (Figure 1(b)).

Cancer metastasis is the primary cause of cancer-related fatal damage to patients. Generally, tumor metastasis occur via bloodstream to distant organs, while most epithelial cancers metastasis firstly spread via lymphatic vessels to their draining lymph node (LN) [23]. Therefore lymphatic metastasis represents the aggressiveness of epithelial cancers to some extent. To inspect whether the expression of miR-656-3p is related to lymphatic metastasis or not, we divided the CRC patients into with or without lymphatic metastasis and compared the miR-656-3p expression level between these two groups. All the patients lymphatic metastasis evaluation were determined according to international standard. In comparison with the CRC patients without lymphatic metastasis, patients with lymphatic metastasis showed significant lower expression of miR-656-3p with *p* < 0.001 (Figure 1(c)), which suggest that miR-656-3p may related to CRC metastasis.

In order to confirm this significant difference between CRC and normal cancer-free tissues, we further detected the expression level of miR-656-3p in 10 different cell lines including normal colonic epithelial NCM460 cell line and some colorectal cancer cell lines, such as SW480, RKO, DLD1, SW48, HCT116, LOVO, HT29, HCT8, and SW620. qRT-PCR was applied on total RNAs extracted from these cell lines. All the colorectal cancer cell lines exhibited significantly lower expression of miR-656-3p than normal colonic epithelial NCM460 cells with *p* < 0.01 and 0.001 (Figure 1(d)). Compared with other colorectal cancer cells, SW480 showed the highest miR-656-3p expression level and DLD1 showed the lowest miR-656-3p expression level, so SW480 was applied for the further miR-656-3p inhibition study and DLD1 for the overexpression study. The low expression level of miR-656-3p in both CRC patients and cell lines may provide valuable information to better understand the mechanism of CRC genesis, and the lower expression of miR-656-3p in CRC patients with lymphatic metastasis suggest that miR-656-3p expression level could be valuable to assess the metastasis of CRC cancer. Meanwhile, the levels of miR-656-3p expression from normal and without LN metastasis tissues were analyzed. Significant higher levels of miR-656-3p was observed in the normal tissues (Figure 1(e)).

### 3.2 miR-656-3p expression inhibits the migration and invasion of colorectal cancer cells in vitro

In order to identify the detailed function of miR-656-3p in CRC metastasis, *in vitro* cell culture system was employed accompanying with miR-656-3p inhibitor and mimics recombined lentivirus transfection approach, such as SW480 and DLD1 cell lines. Three recombined inhibitors vectors including inhibitor #1, #2, and #3 and three recombined mimics vectors including mimics #1, #2, and #3 were constructed, their corresponding empty vectors including INC and mNC were set as control group, respectively. Based the basal miR-656-3p expression level, INC and inhibitor #1, #2, and #3 were transfected to SW480 to establish control and miR-656-3p knockdown stable cell lines, mNC and mimics #1, #2, and #3 were transfected to DLD1 to establish control and miR-656-3p overexpression stable cell lines. Neomycin was used for the selection of transfected cell lines.

**Figure 2.**
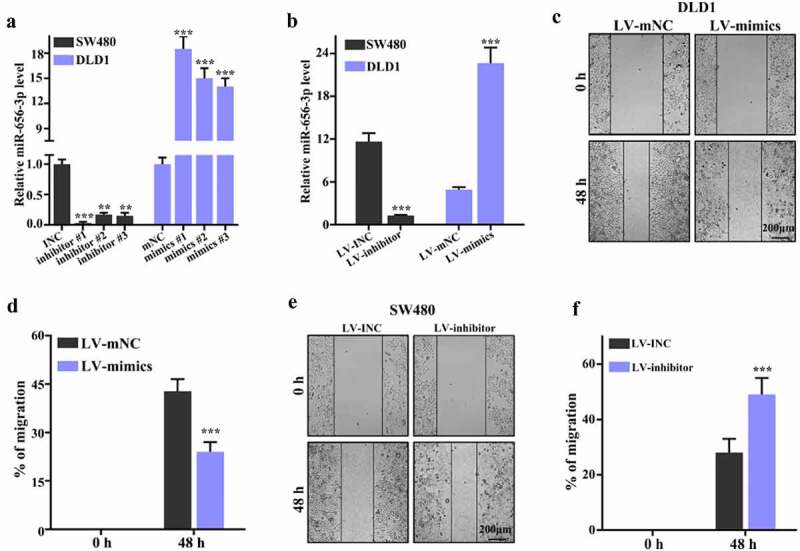
miR-656-3p promotes proliferation of CRC cells *in vitro*. (a) qRT-PCR analysis of miR-656-3p knockdown SW480 cells and miR-656-3p overexpressed DLD1 cells. (b) Inhibitor 1# in SW480 cells and mimics 1# in DLD1 cells were further identified. (c–d) Cell proliferation analysis of miR-656-3p overexpressed DLD1 cells. (e–f) Cell proliferation analysis of miR-656-3p knockdown SW480 cells. **p < 0.01, ***p < 0.001 compared with group INC or LV-INC.

qRT-PCR was applied to verify the transfection effect of miR-656-3p inhibitors and mimics. In SW480 cells, the relative miR-656-3p expression in inhibitor #1, #2, and #3 transfected groups presented significant decrease than that of INC control group (*p* < 0.001, 0.01, and 0.01, respectively), among these inhibitor #1 showed the best inhibition effect (Figure 2(a,b)). In DLD1 cells, the relative miR-656-3p expression in mimics #1, #2, and #3 transfected groups displayed significant increase than that of mNC control group (all *p* < 0.001), and mimics #1 presented the best overexpression effect (Figure 2(a,b)). Therefore, inhibitor #1 and mimics #1 transfected groups were used for further migration study.

Scratch-wound healing was utilized to inspect cells migration under the condition of overexpressing miR-656-3p in DLD1 cells (Figure 2(c)) and lacking miR-656-3p in SW480 cells (Figure 2(e)). The wound was generated on plates with 100% confluency of SW480 and DLD1 stable cell lines. After 48 h growth when generated wound, the migration rates of DLD1 cells treated with constructs of miR-656-3p mimics #1 showed significant decrease than mNC control group (*p* < 0.001, Figure 2(d)), the miR-656-3p overexpression cells presented much slow recovery. In contrast, the migration rates of SW480 cells transfected with miR-656-3p inhibitor #1 exhibited significant increase than INC control group (*p* < 0.001, Figure 2(f)), the miR-656-3p knockdown cells displayed much better recovery. The migration inhibition or promotion phenomenon induced by miR-656-3p were further approved by Transwell (without Matrigel) analysis.

**Figure 3.**
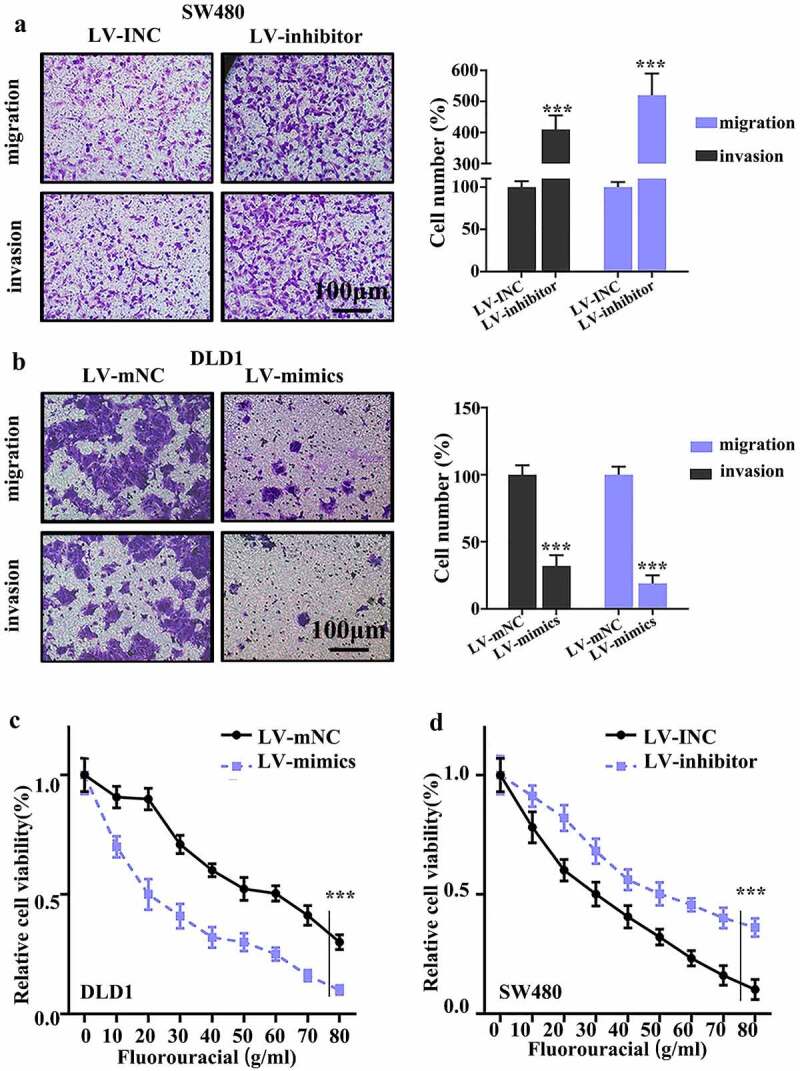
Regulating function of miR-656-3p on CRC cell migration and invasion. (a–b) Cell migration and invasion analysis of miR-656-3p knockdown SW480 cells and miR-656-3p overexpressed DLD1 cells, respectively. (c–d) Cell survival analysis of the 5-FU in miR-656-3p knockdown SW480 cells and miR-656-3p overexpressed DLD1 cells, respectively. ***p < 0.001 compared with group LV-INC or LV-mNC.

Migration inspection was applied with an approach using Transwell without Matrigel. After 48 hours culture, the migration rate of SW480 cells transfected with miR-656-3p inhibitor transporting from top layer to bottom layer exhibited significant increase compared to that of cells transfected with INC control construct (*p* < 0.001, Figure 3(a)). It suggest that the lack of miR-656-3p results in strong significant activity on cell migration in SW480 cell lines. By contrast, the migration rate of DLD1 cells transfected with miR-656-3p mimics construct transporting to bottom layer displayed significant decrease in comparison with control cells transfected with mNC construct (*p* < 0.001, Figure 3(b)), which suggest that the abundance of miR-656-3p results in strong significant defect on cell migration with much slower rate in DLD1 cell lines.

Besides of cells migration inspection, cells invasion inspection was also applied with the manufacturer’s approach of Cultrex 24-well BME Cell Invasion Assay kit. The cells grew on top layer with Matrigel under standard condition, after 48 hours culture, these top layer cells invaded through the Matrigel to bottom layer. The bottom cells number were stained and counted for further comparison. Consistent with the migration inspection results, the miR-656-3p knockdown SW480 cells displayed much higher invasion rate than control cells (*p* < 0.001, Figure 3(a)), while the miR-656-3p overexpression DLD1 cells presented much slower invasion rate than control cells (*p* < 0.001, Figure 3(a)). All the cells migration and invasion data *in vitro* suggest that miR-656-3p may play the inhibition function in the migration and invasion of colorectal cancer.

### 3.3 Knockdown of miR-656-3p promotes the chemo-resistance of colorectal cancer cells

Colorectal cancer as the third most common cancer in the world, the curative therapy is surgery, especially in early tumor stages. While when loco-regional or distant invasion occur, chemotherapy will be introduced. Generally, 5-Fluorouracil (5‐FU) is the main chemotherapy for colorectal cancer at advanced stage or high risk of recurrence. However, the resistance to 5‐FU still exists in some CRC patients, which presented a lower 5‐year survival rate than other CRC patients.

In order to detect the function of miR-656-3p in chemoresistance of colorectal cancer cells, different doses of 5-FU were used in treating SW480 and DLD1 cells transfected with control, miR-656-3p inhibitor, or mimics. MTT was applied for further cell viability analysis, and the absorbance of different group cells were documented at 570 nm. The results showed that compared with mNC control group, the DLD1 cells overexpressed with miR-656-3p exhibited poorer cell viability rate (*p* < 0.001, Figure 3(c)), and the SW480 miR-656-3p knockdown cells displayed better cell viability rate (*p* < 0.001, Figure 3(d)). These difference of cell viability rate in miR-656-3p overexpression or knockdown CRC cells compared with their control cells suggest that miR-656-3p may promote the sensitivity of CRC cells to 5-FU, defect of miR-656-3p could promotes the chemo-resistance of CRC cells.

### 3.4 SGPP1 as a target gene is regulated by miR-656-3p in colorectal cancer cells

**Figure 4.**
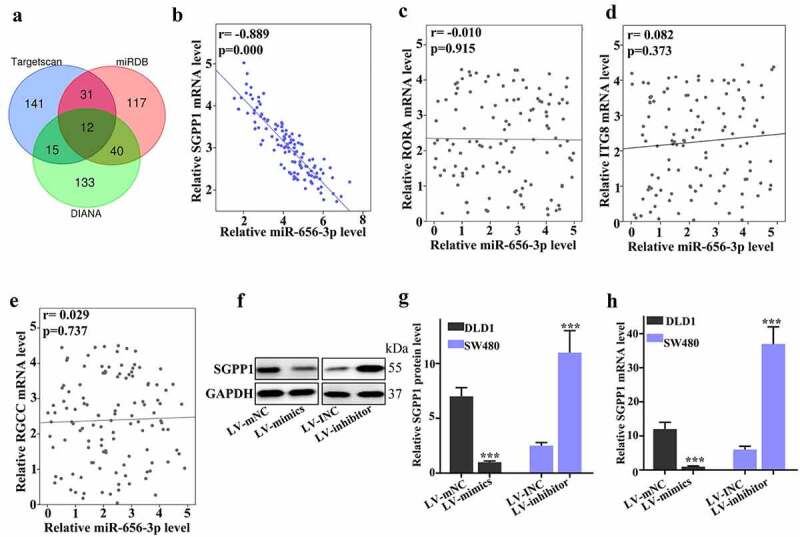
miR-656-3p regulates CRC cell migration and chemotherapy resistance through the SGPP1. (a) Venn diagram analyzed the potential target genes of miR-656-3p in three databases. (b–e) Correlation analysis of miR-656-3p abundance and the mRNA of SGPP1, RORA, ITGB8, and RGCC, respectively. (f–g) WB of SGPP1 protein expression in miR-656-3p knockdown SW480 cells and miR-656-3p overexpressed DLD1 cells. (h) qRT-PCR analysis of SGPP1 protein expression in miR-656-3p knockdown SW480 cells and miR-656-3p overexpressed DLD1 cells. ***p < 0.001 compared with group LV-mNC.

To further predict the target genes of miR-656-3p in CRC cells, three databases including Targetscan, miRDB and DIANA were used in this study. We got 199 potential target genes from Targetscan, 200 from miRDB and 200 from DIANA, respectively. The Venn diagram was performed to overlap these potential target genes predicted from the three databases, and we got 12 genes existed in all the three databases (Figure 4(a)). Based on the biological function, four genes were selected for further analysis, containing of *SGPP1, RORA, ITGB8*, and *RGCC*.

In order to identify the correlation of miR-656-3p and selected four genes (*SGPP1, RORA, ITGB8*, and *RGCC*), the mRNA expression level of miR-656-3p, *SGPP1, RORA, ITGB8*, and *RGCC* in 119 CRC patient tissues were detected by qRT-PCR, and U6 was set as the internal reference. Then the correlation analysis of miR-656-3p and *SGPP1, RORA, ITGB8, and RGCC* were performed separately. The analysis data showed that the relative mRNA level between miR-656-3p and RORA didn’t exhibit any correlation with *r* = 0.010 and *p* = 0.915 (Figure 4(c)). The relative mRNA level of *ITGB8* and *RGCC* were the same, which did not show any correlation with miR-656-3p (*r* = 0.0.082 and 0.029, *p* = 0.373 and 0.737, respectively) (Figure 4(d,e)). However, different from these three target genes, the relative mRNA expression of *SGPP1* presented significantly strong correlation to miR-656-3p with *r* = 0.889 and *p* = 0.000 (Figure 4(b)), which suggest that *SGPP1* could be a target gene modulated by miR-656-3p in CRC cells.

To confirm the correlation between *SGPP1* and miR-656-3p, we inspected the protein and mRNA levels of *SGPP1* in miR-656-3p knocked down SW480 cells and miR-656-3p overexpressed DLD1 cells. Western blot was employed to detect the protein expression of SGPP1 and qRT-PCR was for the detection of mRNA expression of *SGPP1*. In DLD1 cells, compared with mNC control group, miR-656-3p overexpressed group displayed obviously downregulation of SGPP1, both in protein level (*p* < 0.001, Figure 4(f,g)) and mRNA level (*p* < 0.001, Figure 4(h)). Corresponding to that, in SW480 cells, the expression level of SGPP1 in miR-656-3p knocked down group presented significantly upregulation in comparison with that of INC control group, and also both in protein level (*p* < 0.001, Figure 4(f,g)) and mRNA level (*p* < 0.001, Figure 4(h)). These protein and mRNA inspection of SGPP1 *in vitro* CRC cell lines further supported that SGPP1 is inhibited by miR-656-3p as a downstream target gene in CRC cells.

### 3.5 SGPP1 is a risk factor of colorectal cancer through bioinformatics analysis

**Figure 5.**
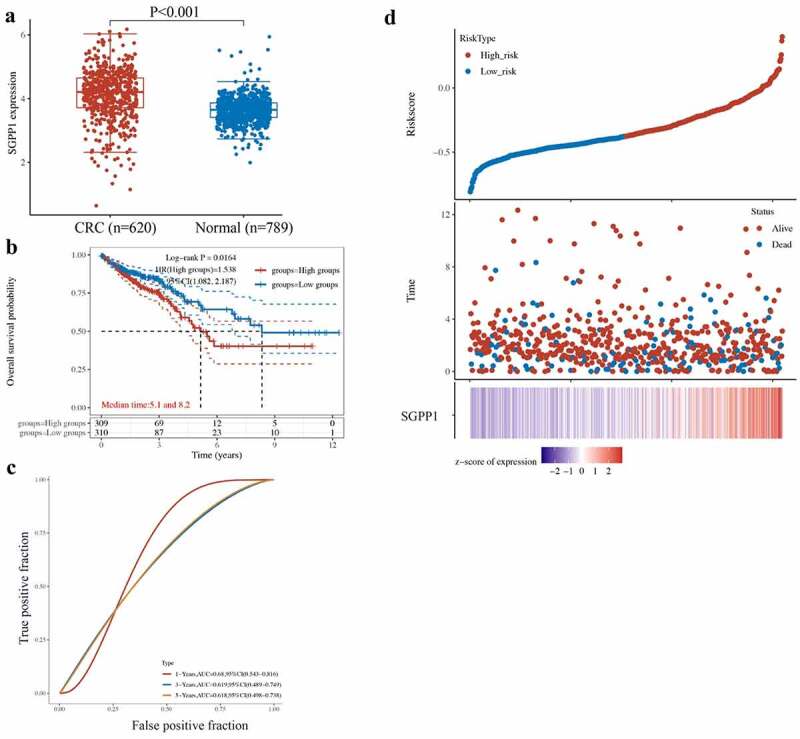
*SGPP1 is a risk factor of* colorectal cancer. (a) The expression of SGPP1 in CRC and normal patients were analyzed using TCGA database. (b) High expression of SGPP1 indicates low overall survival probability. (c) SGPP1 could be a potential predictor of CRC. (d) High expression of SGPP1 suggests high risk of CRC.

Through bioinformatics analysis, we found that the SGPP1 expression was greatly increased in the CRC patients (Figure 5(a)). In addition, high expression level of SGPP1 indicates remarkable decrease of overall survival probability (Figure 5(b)). The 1-year – AUC of true positive fraction is 0.68, indicating that SGPP1 is a potential risk factor of CRC (Figure 5(c)). Meanwhile, High expression of SGPP1 indicates a high risk of CRC (Figure 5(d)).

### 3.6 miR-656-3p modulate SGPP1 by directly binding to 3’ UTR of SGPP1

**Figure 6.**
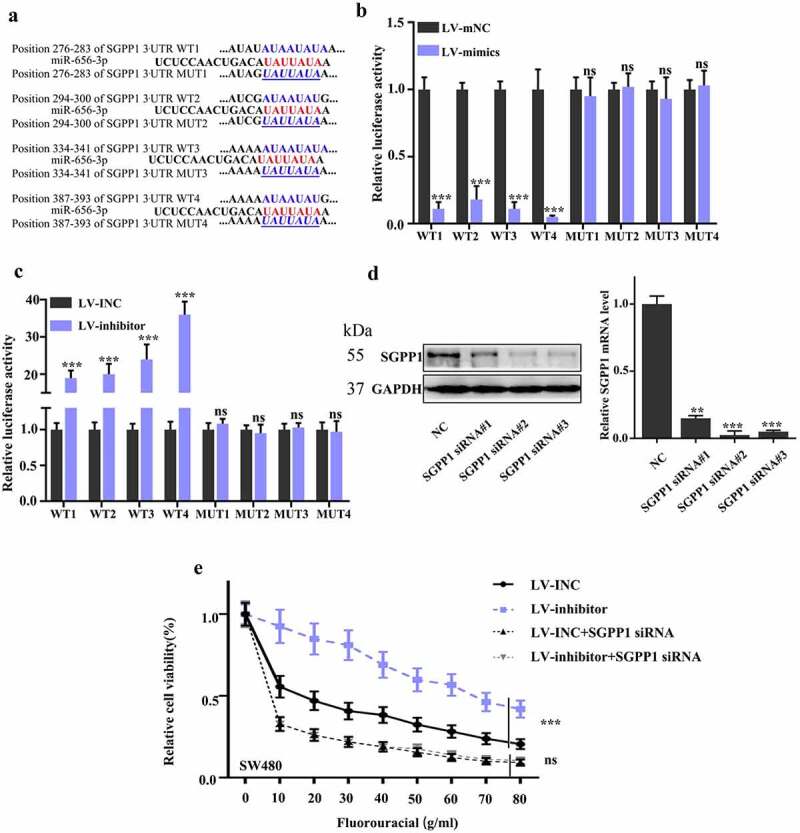
miR-656-3p directly binds to 3’ UTR of SGPP1 mRNA. (a) Potential binding sites between miR-656-3p and 3’ UTR of SGPP1 mRNA. (b–c) Dual fluorescein reporter gene analysis of the binding sites between miR-656-3p, wild type, and mutation of the 3’ UTR in SGPP1 mRNA. (d) Protein and mRNA analysis of the SGPP1 knockdown effect in SW480 cells. (e) Cell survival analysis of the chemotherapy drugs (5-FU) in miR-656-3p and SGPP1 knockdown-treated SW480 cells. ***p < 0.001 compared with group LV-mNC or LV-INC.

According to above result, we got that *SGPP1* is modulated by miR-656-3p in CRC cells. Generally, micro RNA regulate gene expression through complementary base pairing with 3’ UTR of mRNA, resulting in mRNA degradation or inhibition, therefore blocking the final translation [24]. Based on the characteristic of micro RNA, we aligned the sequence of miR-656-3p and 3’ UTR of *SGPP1*, and found out four potential binding sites between miR-656-3p and 3’ UTR of *SGPP1* (Figure 6(a)). Dual fluorescein reporter gene analysis were performed for the further detection of miR-656-3p binding sites on *SGPP1*.

These 3’ UTR of *SGPP1* with potential binding sites were then constructed to renilla fluorescein reporter vector as wild-type group, such as WT1, WT2, WT3, and WT4. Meanwhile, the sequence in these four binding sites were mutated and the corresponding 3’ UTR of *SGPP1* with these mutant were also constructed to renilla fluorescein reporter vector as mutant group, such as MUT1, MUT2, MUT3, and MUT4. All the constructs were transfected to miR-656-3p overexpressed DLD1 cells and miR-656-3p knocked down SW480 cells, and then the activation of luciferase were detected.Comparing with mNC control cells, the relative luciferase activity of wild-type group (WT1-4) significantly decreased in miR-656-3p overexpression DLD1 cells (all *p* < 0.001), while the mutant group didn’t show any difference (Figure 6(b)). In contrast, in miR-656-3p knocked down SW480 cells, the relative luciferase activity of wild type group (WT 1–4) obviously increased compared to that of INC control cells, and the mutant group still didn’t present any difference (Figure 6(c)). These suggest that miR-656-3p indeed could bind to these four binding sites (WT1-4) of *SGPP1*, and once these binding sites were mutated, the binding also disappeared.

To further explore the detailed mechanism of *SGPP1* in CRC cells, we designed the siRNA (#1, #2, and #3) of *SGPP1*. By Western blot and qRT-PCR, we verified the knockdown effect of *SGPP1* siRNA (#1, #2 and #3). In comparison with NC control, the protein expression is significantly decreased in miR-656-3p knocked down SW480 cells when transfected with siRNA #1, #2, and #3 (Figure 6(d) left), and same with the mRNA expression level (*p* < 0.01 and 0.001, Figure 6(d) right). Based on the knockdown effect, *SGPP1* siRNA #2 was selected for the further study. The increased chemo-resistance in CRC miR-656-3p defect cells have been identified above, and to examine whether SGPP1 participates in this process, we detected the chemo-resistance of CRC miR-656-3p defect cells and CRC miR-656-3p/SGPP1 defect cells. As we have seen before, the chemo-resistance is significantly promoted in miR-656-3p knocked down SW480 cells compared with that of INC control cells, but the sensitivity to chemotherapy drugs (5-FU) could be dramatically recovered when *SGPP1* was knocked down in miR-656-3p defect SW480 cells (Figure 6(e)). The defect of *SGPP1* in SW480 cells increased the cells sensitivity to chemotherapy drugs (5-FU), both in INC control and miR-656-3p knocked down cells, and the increased chemo-resistance in miR-656-3p defect SW480 cells was even recovered to the same sensitivity level with INC control cells (Figure 6(e)).

Taken together, the downregulated miR-656-3p in both CRC patients may provide valuable information to exploring the mechanism of CRC genesis, and the higher expression of miR-656-3p in CRC patients without lymphatic metastasis than with lymphatic metastasis suggest that miR-656-3p may play the inhibition function in the migration and invasion of colorectal cancer, which was also further approved in *vitro* CRC cells culture system. miR-656-3p may also promote the sensitivity of CRC cells to chemotherapy drugs (5-FU), and the defect of miR-656-3p in CRC cells could promotes cells chemo-resistance. *SGPP1* is the target gene inhibited by miR-656-3p in CRC cells, and miR-656-3p modulate *SGPP1* by directly binding to the 3’ UTR of *SGPP1. SGPP1* participates the process of cells chemo-resistance, the knockdown of *SGPP1* in CRC cells could increase cells sensitivity to chemotherapy drugs (5-FU). The expression of miR-656-3p and its target gene *SGPP1* could provide valued information to diagnose, assess the metastasis, and chemotherapy for CRC cancer.

### 3.7 Knockdown of SGPP1 greatly suppressed tumor growth in vivo

**Figure 7.**
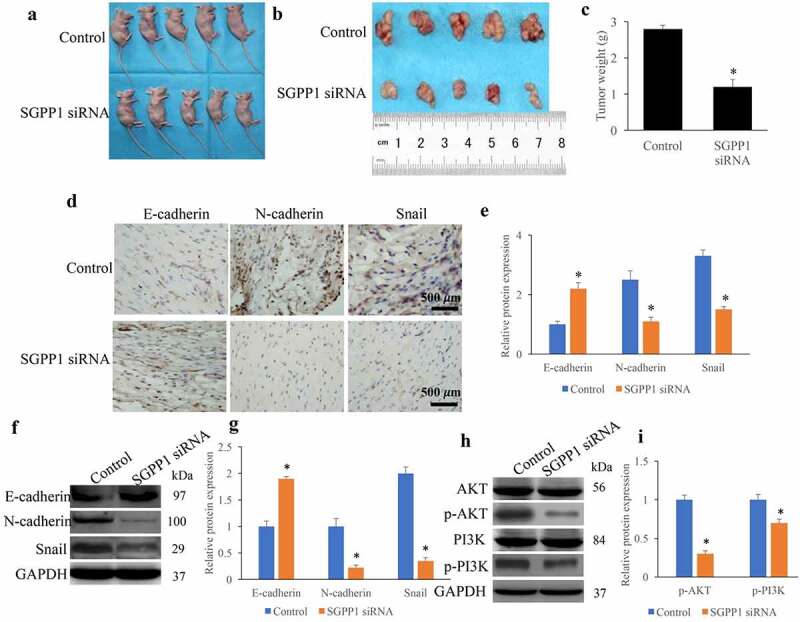
SGPP1 mRNA significantly suppressed the growth of tumor in vivo. (a) Representative animal images transplanted with tumor cells. (b) Representative tumors isolated from mice. (c) The tumor size was quantitatively analyzed. (d) The expression of EMT related proteins in tumor tissues were measured through IHC. (e) The levels of EMT-related proteins were quantitatively analyzed. (f) The expression of EMT related proteins in tumor tissues were measured through Western blotting. (g) The levels of EMT-related proteins were quantitatively analyzed. (h) The protein levels of p-AKT and p-PI3K were measured by Western blotting. (i) The protein levels of p-AKT and p-PI3K were analyzed. *p < 0.05 compared with group control.

The transplanted tumor model was established to investigate the role of SGPP1 siRNA in regulating tumor growth. We found that the tumor size was markedly inhibited after transfecting SGPP1 siRNA (Figure 7(a-c)). In addition, the epithelial mesenchymal transition (EMT) process was remarkably inhibited by SGPP1 siRNA through increasing E-cadherin, but decreasing N-cadherin and Snail (Figure 7(d-f)). The protein levels of p-AKT and p-PI3K were measured. We found that SGPP1 siRNA significantly suppressed the levels of p-AKT and p-PI3K compared with group control (Figure 7(h-i)).

## 4. Discussion

Colorectal cancer is one of the malignant tumors with the highest prevalence of gastrointestinal system. It is noticed that the incidence of CRC has shown an upward trend in China year by year [25,26] along with the increase of industrialization from urbanization. But the etiology of CRC is not yet fully illustrated. Investigations found that the incidence of CRC is related with presence of intestinal polyposis, stress and inflammation, and a high risk family history of cancer [27]. Invasion and migration of advanced CRC were recognized as the main reason for death of CRC patients [27,28]. The improvement of diagnostic techniques and clinic treatments including surgery, chemotherapy, and radiotherapy can promote the early detection and improve the overall survival of the patients. Up to date, the most current treatment options are always determined based on the classic clinic-pathological characteristics and stage of cancer in absence of novel tumor markers, which is particularly for early diagnosis and personalized treatment for CRC patients.

miR-656-3p is a conserved small RNA molecule widely distributed in colon tissues. Previous data approved that miR-656-3p can affect the occurrence and development of CRC with unclear molecular mechanism [29]. In this study, our evidences suggested that the modification of miRNA-656-3p expression was linked to the cell proliferation and chemo-resistance of CRC in both in vitro and patients. Our data indicates that the miRNA-656-3p may function as a potential target of CRC for practical treatments. As the complicated process, the underlying mechanism of miRNA-656-3p targeting on CRC is holding a strong potential for unveiling tumorigenesis and development of CRC. Overexpression of miRNA-656-3p or knockdown of miRNA-656-3p had a major function in the proliferation, migration, invasion, and chemo-resistance of CRC. The dysfunction of miRNA-656-3p could be brutal causes interfering the progression of CRC.

As a subtype of S1P phosphorylase (SGPPs), SGPPs has two subtypes including SGPP1 and SGPP2 [18]. With distribution of the endoplasmic reticulum, they play a role mainly by regulating the phosphorylation state of sphingosine-1-phosphate (S1P) intracellularly and extracellularly [19]. As a second messenger in the cell, sphingolipid S1P regulates multiple cell functions such as differentiation, proliferation, metastasis, cytoskeleton remodeling, aging, and apoptosis. In tumorigenesis, SGPP1 is a tumor suppressor gene and intensively involved in the invasion and metastasis of a variety of tumors [17]. In gastric cancer tissues, the SGPP1 expression is downregulated compared to that in adjacent and cancer-free tissues. A positive connection between the low expression of SGPP1 and lymph node and distant metastasis of gastric cancer were confirmed. Another group unveiled that downregulation of SGPP1 can promote the transformation of cells to epidermal growth factor (EGF) migration, and overexpression of SGPP1 can reduce the chemotactic effect of cells on EGF, which means that S1P-associated SGPP1 retains a key role in EGF-directed cell migration [30].

It had been approved that the binding to S1P3-Gαq, S1P promotes activation of Rac1 via p38/ERKs/Akt signaling pathways, and then ultimately induces the transcriptional activation of MMP9. The activated MMP9 participates in the invasion and metastasis in breast cancer with high grade [31]. Furthermore, S1P upregulated MMP2 expression and additional the enhancement of migration ability targeting on endothelial cells. Recently, more comprehensive investigation approved that SGPP can deeply associate with calcium signaling pathway which may provide more effective prospects on unveiling miRNA-656-3p associated SGPP1 mechanism in colorectal cancer [19].

## 5. Conclusion

In this study, our data suggest that the expression of SGPP1 in CRC is closely correlated to miRNA-656-3p in CRC with solid evidences. The miRNA-656-3p associated regulation of SGPP1 expression could be critical for designing earlier treatment and diagnostics in chemotherapy resistance, migration, invasion, and cell proliferation.

## Abbreviations

CRC: Colorectal cancer, cDNA: complementary DNA, EGF: epidermal growth factor, MUC: mucins, dMMR: DNA mismatch repair, dsRNA: double-stranded siRNAs, FBS: fetal bovine serum, 5-FU: 5-fluorouracil, LN: lymph node ncRNA: MMP9: matrix metallopeptidase 9, ncRNA: noncoding RNA, miRNA: micro RNA, PDCD4: programmed cell death 4, PTEN: phosphatase and tensin homolog, RLU: renilla luciferase, RT-PCR: real-time reverse transcription-polymerase chain reaction, S1P: sphingosine-1-phosphate, SDS-PAGE: Sodium dodecylsulfate–polyacrylamide, SGPP: S1P phosphorylase, TPM1: target and regulate tropomyosin 1
